# A novel web-based dynamic prognostic nomogram for gastric signet ring cell carcinoma: a multicenter population-based study

**DOI:** 10.3389/fimmu.2024.1365834

**Published:** 2024-04-10

**Authors:** Yujuan Jiang, Haitao Hu, Xinxin Shao, Weikun Li, Yiming Lu, Jianwei Liang, Yantao Tian

**Affiliations:** ^1^ Department of Pancreatic and Gastric Surgery, National Cancer Center/National Clinical Research Center for Cancer/Cancer Hospital, Chinese Academy of Medical Sciences and Peking Union Medical College, Beijing, China; ^2^ Department of Colorectal Surgery, National Cancer Center/National Clinical Research Center for Cancer/Cancer Hospital, Chinese Academy of Medical Sciences and Peking Union Medical College, Beijing, China

**Keywords:** gastric signet ring cell carcinoma, prognosis, dynamic nomogram, overall survival, cancer-specific survival

## Abstract

**Background:**

Gastric signet ring cell carcinoma (GSRCC) is a rare and highly malignant disease with a poor prognosis. To assess the overall survival (OS) and cancer-specific survival (CSS) of patients with GSRCC, prognostic nomograms were developed and validated using common clinical factors.

**Methods:**

This retrospective cohort study included patients diagnosed with GSRCC between 2011 and 2018 from the National Cancer Center (n = 1453) and SEER databases (n = 2745). Prognostic nomograms were established by identifying independent prognostic factors using univariate and multivariate Cox regression analyses. The calibration curve and C-index were used to assess the predictions. The clinical usefulness of the survival prediction model was further evaluated using the DCA and ROC curves. The models were internally validated in the training cohort and externally validated in the validation cohort. Two web servers were created to make the nomogram easier to use.

**Results:**

Patients with GSRCC were divided into training (*n* = 2938) and validation (*n* = 1260) cohorts. The nomograms incorporated six predictors: age, race, tumor site, tumor size, N stage, T stage, and AJCC stage. Excellent agreement was observed between the internal and exterior calibration plots for the GSRCC survival estimates. The C-index and area under the ROC curve were roughly greater than 0.7. Both nomograms had adequate clinical efficacy, as demonstrated by the DCA plots. Furthermore, we developed a dynamic web application utilizing the constructed nomograms available at https://jiangyujuan.shinyapps.io/OS-nomogram/ and https://jiangyujuan.shinyapps.io/DynNomapp-DFS/.

**Conclusion:**

We developed web-based dynamic nomograms utilizing six independent prognostic variables that assist physicians in estimating the OS and CSS of patients with GSRCC.

## Introduction

Gastric cancer (GC) ranks fifth in incidence and fourth in cancer-related mortality, leading to approximately 768,793 deaths annually (1). Gastric signet ring cell carcinoma (GSRCC) is a unique type of GC characterized by abundant mucus, with the nucleus pushed to the side by intracytoplasmic mucin, representing 35–45% of new adenocarcinomas (2). Due to the underdevelopment of screening technologies in previous years, the majority of GSRCC patients were diagnosed with an advanced disease. The prognosis of GC patients has improved due to recent advancements in surgery, chemotherapy, radiation, targeted therapy, and immunotherapy; still, the 5-year survival rate of GSRCC is only about 32.1% (3). Notably, the biological behavior of GSRCC is significantly heterogeneous compared to that of non-GSRCCs, which can be attributed to the depth of tumor infiltration (4). GSRCC is typically an advanced tumor stage that is resistant to chemotherapy (2). Radical tumor resection (R0) is the most effective treatment for GSRCC. However, the R0 resection rate of GSRCC was significantly lower than that of non-GSRCC (56.0% vs. 74%, *P *= 0.019), and the postoperative peritoneal recurrence rate was much higher than that of non-GSRC (52.2% vs. 21.4%) (2). Hence, there is a need to improve the postoperative clinical outcomes of patients with GSRCC through individualized treatment.

Tumor-node-metastasis (TNM) staging is currently used to regularly estimate the prognosis of GC patients. However, due to the considerable genetic heterogeneity in GC, recurrence and mortality may differ significantly even among GC patients with similar TNM stage. Nomograms, which are commonly used for assessing cancer patient prognosis and personally predicting survival rates, are more suitable for clinical patient management than the TNM staging system. However, despite the establishment of several postoperative nomograms that have significantly contributed to the management of patients with GSRCC, some issues have arisen (5–8). First, these models focused solely on the overall postoperative survival and did not predict cancer-specific survival. Second, calibration tests, external model validations, and evaluations of the clinical usefulness of these models are lacking, making it difficult to assess their accuracy and practicability. Moreover, the traditional predictive models are not sufficiently simple. A web-based dynamic nomogram that calculates the probability of a disease is a more precise and practical tool than standard nomograms and some predictive models. To classify the prognosis of GSRCC patients, it is thus essential to create a straightforward, user-friendly, and reliable prognosis prediction model.

This study established two new postoperative web-based nomograms for patients with stage I-III GSRCC to predict 1-, 3-, and 5-year overall survival (OS) and cancer-specific survival (CSS).

## Materials and methods

### Study population

**Figure 1:**
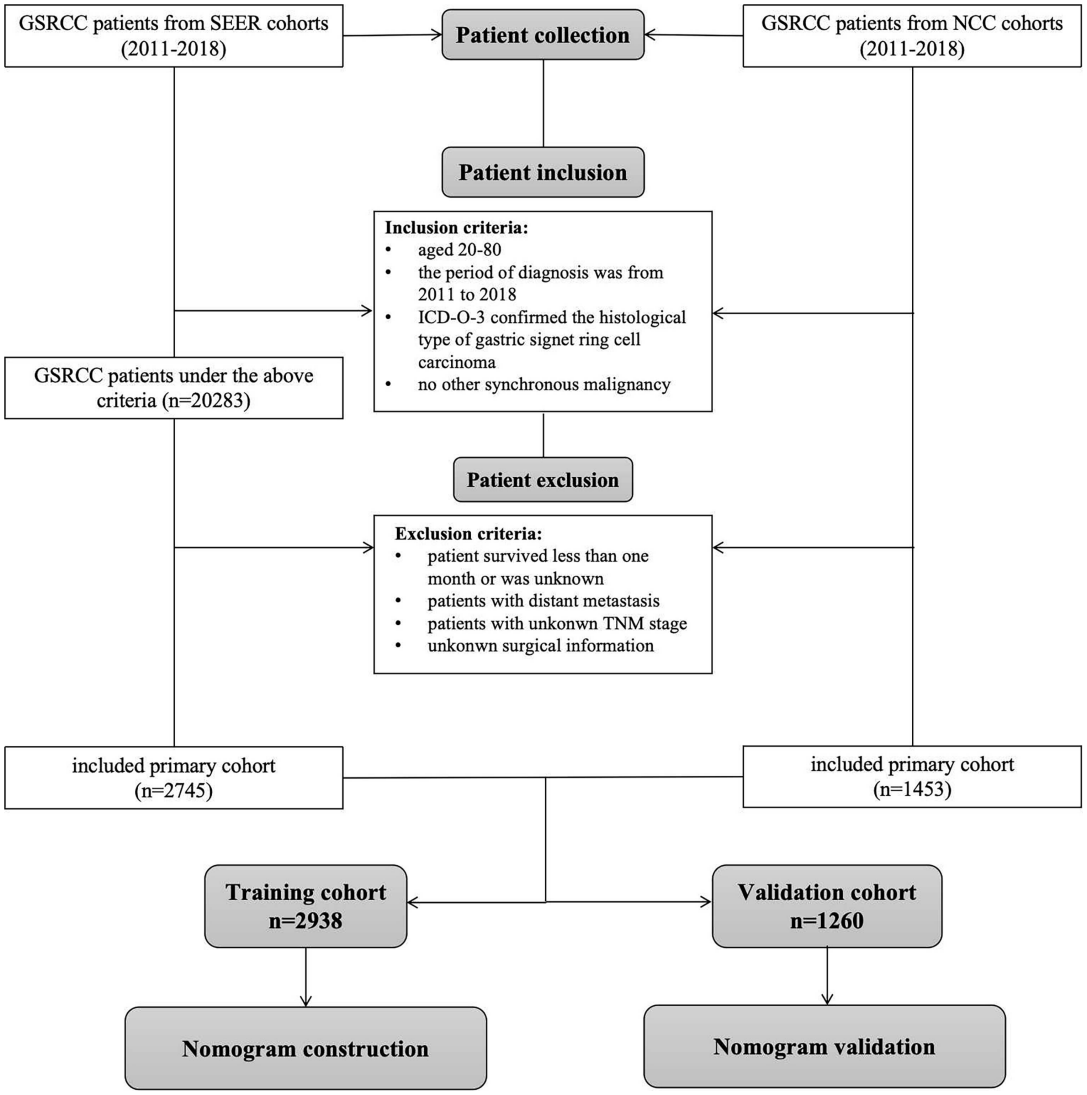
Flow chart of the construction and validation of the nomogram models.

Patients diagnosed with GSRCC between 2011 and 2018 from the Surveillance, Epidemiology, and End Results (SEER) database (http://www.seer.cancer.gov) were identified using the SEER*Stat software (version 8.4.2). The eligibility criteria were as follows: (1) ICD-O-3 histology code of 8490, (2) site code of C16.0–16.9, (3) lack of other synchronous malignancies, (4) age between 20 and 80 years, and (5) radical surgical treatment. The exclusion criteria were as follows: (1) patients who did not undergo radical surgery, (2) patients who survived less than one month or had an unknown survival status, (3) patients with distant metastasis, and (4) patients with unclear clinicopathological characteristics such as TNM stage. Patients diagnosed with GSRCC between 2011 and 2018 at the National Cancer Center (NCC cohort) were included based on the above criteria. The patient screening process is illustrated in **Figure 1**. Ethical approval was obtained from the Ethics Committee of the National Cancer Center/Cancer Hospital, the Chinese Academy of Medical Science, and Peking Union Medical College (NCC2023C-657). Due to the retrospective nature of this study, informed consent was not required.

### Prognostic variables

Patient variables such as age, sex, race, tumor size, tumor site, grade, AJCC TNM stage, pathological N stage, pathological T stage, survival time, and status were collected. All patients underwent restaging based on the criteria outlined in the AJCC on Cancer 8th edition staging manual. OS was the primary endpoint and was determined from the date of diagnosis until death from any cause or last follow-up. CSS was the secondary endpoint and was selected from the date of diagnosis until GSRCC cancer-related death or last follow-up. In the SEER cohort, CSS is defined by the SEER cause-specific death category. In the NCC cohort, we carefully monitored every patient in the after surgery, and we included those who lost their lives due to GSRCC in the CSS survival analysis. Patients were categorized into two age groups: young (<60 years) and old (>60 years). The patient groups were stratified based on tumor size: <5 cm and >5 cm.

### Establishment and validation of the nomogram

The patients were randomly assigned to the training (70%) and validation (30%) cohorts. Independent prognostic factors were chosen using a backward stepwise method with AIC to minimize information loss. Subsequently, a univariate Cox proportional hazard regression analysis was performed for the training cohort. Factors associated with a P value < 0.1 according to the univariate analysis were subsequently included in the multivariate Cox analysis to determine the hazard ratio (HR) and p-value for each independent prognostic variable. The factors with P <0.05 in COX multivariate regression analyses were considered independent risk factors for prognosis, and the prognostic prediction models were constructed on the basis of these factors. When building a multivariate regression model, we derive the regression coefficient β for each variable. The nomogram scores the variable with the largest regression coefficient in the regression model as 100 points. All other variables are converted using it as a criterion, e.g. β=2 with a score of 100. If the other variables β=1, the score is 1*100/2. After converting the regression coefficients to a 0–100 point scale based on multivariate analysis, we developed two prognostic nomograms to predict OS and CSS in patients with GSRCC using independent predictive variables. The accuracy of the nomograms was validated internally and externally using calibration curves. The C-index and area under the ROC curve (AUC) were used to evaluate the discriminative ability. To evaluate the models’ clinical applicability, decision curve analysis (DCA) was used to calculate the net benefit at various threshold probabilities. The net benefit is defined as that true positive minus false positive. To put it succinctly, all patient death curves and none patient death curves were drawn as two references. DCA calculates the clinical benefit compared with the reference lines. The higher the net benefit, the more practical and effective the prediction model is in clinical practice. The patients were categorized into low- and high-risk groups based on the risk score calculated for each patient. Finally, Kaplan–−Meier plots were generated to assess potential disparities in overall survival between the high- and low-risk patient groups.

### Statistical analysis

Categorical variables are presented as numbers and percentages. Pearson’s correlation analysis was used to ensure that there was no collinearity between the screened variables (**Supplementary Figure 2**). Univariable and multivariable Cox proportional hazards models were used to analyze DFS and OS, in which hazard ratios (HRs) and their 95% confidence intervals (CIs) were calculated. Survival analysis was performed using the Kaplan–−Meier method and log-rank test. A two-tailed significance level of P < 0.05 (two-tailed) was used for all statistical tests. Statistical analysis was conducted using R (version 4.0.2 (https://www.r-project.org/). The “caret” package was used to partition the training and validation cohorts randomly. Dynamic nomogram models were constructed with the “rms” and “Dynnom” packages.

## Results

### Clinicopathological characteristics of patients

A total of 4198 patients diagnosed with GSRCC between 2011 and 2018 were included in this study and were randomly divided into two cohorts: the training cohort (n = 2938; 70%) and the validation cohort (n = 1260; 30%). Comprehensive descriptive statistics are presented in **Table 1**. Most patients were white (n = 1883; 44.9%) or male (n = 2416; 57.5%). More than half of the patients (51.0%) were in the older age group. Moreover, 2277 patients (54.2%) had a tumor size of ≤5 cm. The predominant grade was III (n =3423, 81.5%). Moreover, 1857 patients (44.2%) underwent surgery, 1126 (26.8%) underwent surgery plus chemotherapy, and 821 (19.6%) underwent surgery with neoadjuvant therapy. The gastric antrum, encompassing the pylorus, was the most common site of GSRCC (32.1%). The median follow-up duration for all patients was 45 months (IQR, 25–69 months).

**Table 1:** The demographic and clinical characteristics of the patients with GSRCC in the SEER and NCC cohorts.

Factor	Total cohort (n = 4198)	SEER cohort (n = 2745)	NCC cohort (n = 1453)
Sex (%)			
Male	2416 (57.5%)	1468 (53.5%)	948 (65.2%)
Female	1782 (42.4%)	1277 (46.5%)	505 (34.8%)
Age			
≤60 years	2057 (49.0%)	1162 (42.3%)	895 (61.6%)
>60 years	2141 (51.0%)	1583 (57.7%)	558 (38.4%)
Race (%)			
Black	331 (7.9%)	331 (12.1%)	0
White	1883 (44.9%)	1883 (68.6%)	0
Chinese	1554 (37.0%)	101 (3.7%)	1453 (100%)
Other	430 (10.2%)	430 (15.7%)	0
Site (%)			
1/3 U	973 (23.2%)	628 (22.9%)	345 (23.7%)
1/3 M	428 (10.2%)	330 (12.0%)	98 (6.7%)
1/3 L	1349 (32.1%)	778 (28.3%)	571 (39.3%)
Curvature	761 (18.1%)	415 (15.1%)	346 (23.8%)
Total stomach	414 (9.9%)	329 (12.0%)	85 (5.9%)
others	273 (6.5%)	265 (9.7%)	8 (0.6%)
Size (%)			
≤5 cm	2277 (54.2%)	1268 (46.2%)	1009 (69.4%)
>5 cm	1921 (45.8%)	1477 (53.8%)	444 (30.6%)
Grade (%)			
I	34 (0.8%)	7 (0.3%)	27 (1.9%)
II	371 (8.8%)	58 (2.1%)	313 (21.5%)
III	3423 (81.5%)	2311 (84.2%)	1112 (76.5%)
IV	66 (1.6%)	65 (2.4%)	1 (0.1%)
NA	304 (7.2%)	304 (11.1%)	0
AJCC stage (%)			
IA	907 (21.6%)	568 (20.7%)	339 (23.3%)
IB	398 (9.5%)	276 (10.1%)	122 (8.4%)
IIA	442 (10.5%)	304 (11.1%)	138 (9.5%)
IIB	580 (13.8%)	400 (14.6%)	180 (12.4%)
IIIA	537 (12.8%)	387 (14.1%)	150 (10.3%)
IIIB	585 (13.9%)	375 (13.7%)	210 (14.5%)
IIIC	749 (17.8%)	435 (15.8%)	314 (21.6%)
pT stage (%)			
T1	1166 (27.8%)	751 (27.4%)	415 (28.6%)
T2	493 (11.7%)	320 (11.7%)	173 (11.9%)
T3	1232 (29.3%)	906 (33.0%)	326 (22.4%)
T4	1307 (31.1%)	768 (28.0%)	539 (37.1%)
pN stage (%)			
N0	1876 (44.7%)	1284 (46.8%)	592 (40.7%)
N1	759 (18.1%)	566 (20.6%)	193 (13.3%)
N2	575 (13.7%)	355 (12.9%)	220 (15.1%)
N3	988 (23.5%)	540 (19.7%)	448 (30.8%)
Therapy (%)			
surgery only	1857 (44.2%)	1664 (60.6%)	193 (13.3%)
surgery plus neo/chemo	392 (9.3%)	172 (6.3%)	220 (15.1%)
surgery plus chemo	1126 (26.8%)	678 (24.7%)	448 (30.8%)
surgery plus neo	821 (19.6%)	229 (8.3%)	592 (40.7%)
NA	2 (0.5%)	2 (0.1%)	0

pN, pathologic N stage; pT, pathologic T stage.

The tumor site was divided into 1/3 U (cardia, fundus, gastroesophageal junction), 1/3 M (body), 1/3 L (antrum, pylorus), curvature, total stomach, and other (gastric remnant, anastomosis, and linitis plastica) parts of the stomach. NA, not applicable.

### Univariate and multivariate analysis of clinicopathological features

A ratio of 7:3 was used to randomly assign the patients to the training and validation cohorts. Preliminary univariate analysis of the training dataset revealed significant correlations (all *P* < 0.1) between OS and several variables including age, race, tumor size, tumor site, depth of invasion, pN, and AJCC stage. Subsequently, the predictive features that exhibited significant associations with OS in univariate analyses were subjected to multivariate Cox proportional hazards regression analysis. Multivariate analysis results indicated that only age (hazard ratio 1.60, 95% CI 1.45-1.76, P < 0.001), race (P < 0.05), tumor site (P < 0.05), tumor size (hazard ratio 1.80, 95% CI 1.62-2.00 P < 0.001), AJCC stage (P < 0.05), and pN stage (P < 0.001) were significantly associated with OS (**Table 2**). A forest plot illustrating the hazard ratios (HRs) and 95% confidence intervals (CIs) for OS based on the Cox proportional hazards regression analysis is presented in **Supplementary Figure 1A**.

**Table 2:** Univariate and multivariate analyses of overall survival in the training cohort.

Factor	Univariate analyses		Multivariate analyses	
	*p* Value	HR (95% CI)	*p* Value	HR (95% CI)
**Sex**	0.143		0.266	
Male		Reference		Reference
Female		0.93(0.84-1.02)		0.94(0.85-1.04)
**Age**	**<0.001**		**<0.001**	
≤60 years		Reference		Reference
>60 years		1.73(1.57-1.90)		1.60(1.45-1.76)
Race				
Black		Reference		Reference
White	0.994	1(0.85-1.18)	**0.030**	0.84(0.72-0.98)
Chinese	**<0.001**	0.34(0.29-0.41)	**<0.001**	0.41(0.34-0.49)
Other	**<0.001**	0.68(0.55-0.83)	**0.001**	0.71(0.57-0.87)
Site				
1/3 U		Reference		Reference
1/3 M	**<0.001**	0.62(0.52-0.75)	0.606	0.95(0.77-1.16)
1/3 L	**<0.001**	0.54(0.48-0.62)	0.387	0.93(0.80-1.09)
Curvature	**<0.001**	0.52(0.45-0.61)	0.280	0.91(0.76-1.08)
Total stomach	**0.010**	1.23(1.05-1.45)	**<0.001**	1.39(1.15-1.67)
others	**<0.001**	1.53(1.28-1.82)	**0.023**	1.28(1.03-1.58)
**Size**			**<0.001**	
≤5 cm		Reference		Reference
>5 cm	**<0.001**	2.85(2.58-3.14)		1.80(1.62-2.00)
Grade (%)				
I		Reference		Reference
II	0.730	1.28(0.31-5.21)	0.985	1.01(0.32-3.23)
III	0.097	3.23(0.81-12.93)	0.461	1.54(0.49-4.80)
IV	0.097	3.36(0.80-14.01)	0.693	1.27(0.39-4.20)
NA	0.064	3.74(0.93-15.06)	0.286	1.87(0.59-5.91)
AJCC stage				
IA		Reference		Reference
IB	**<0.001**	1.57(1.25-1.97)	**<0.001**	1.83(1.35-2.47)
IIA	**<0.001**	1.85(1.49-2.29)	**<0.001**	2.29(1.53-3.41)
IIB	**<0.001**	2.58(2.14-3.11)	**<0.001**	3.50(2.21-5.54)
IIIA	**<0.001**	3.07(2.55-3.70)	**<0.001**	4.22(2.41-7.38)
IIIB	**<0.001**	4.08(3.40-4.89)	**<0.001**	5.78(3.19-10.46)
IIIC	**<0.001**	4.96(4.18-5.89)	**<0.001**	8.04(3.93-16.45)
pT stage				
T1		Reference		Reference
T2	**0.007**	1.31(1.08-1.61)	0.146	0.80(0.59-1.08)
T3	**<0.001**	2.73(2.37-3.14)	0.626	0.91(0.64-1.31)
T4	**<0.001**	3.43(2.98-3.95)	0.389	0.82(0.52-1.29)
pN stage				
N0		Reference		Reference
N1	**<0.001**	1.59(1.39-1.82)	0.095	0.85(0.70-1.03)
N2	**<0.001**	1.58(1.36-1.83)	**0.002**	0.66(0.51-0.86)
N3	**<0.001**	2.42(2.15-2.73)	**0.007**	0.64(0.46-0.89)
Therapy				
surgery only		Reference		
surgery plus neo/chemo	0.115	0.57(0.47-0.68)		
surgery plus chemo	0.322	0.87(0.78-0.97)		
surgery plus neo	0.979	1.00(0.82-1.21)		
NA	0.751	1.25(0.31-5.01)		

pN, pathologic N stage; pT, pathologic T stage; CI, confidence interval; HR, hazard ratio.

Bold represents significant statistical difference (P<0.05).

NA, not applicable.

In the training cohort, univariate analysis revealed that age, race, tumor size, tumor site, invasion depth, pN stage, and AJCC stage were associated with CSS in patients with GSRCC. Subsequent multivariate analysis identified age (hazard ratio 1.46, 95% CI 1.31-1.62, P < 0.001), race (P < 0.05), tumor site (P < 0.05), tumor size (hazard ratio 1.93, 95% CI 1.72-2.16, P < 0.001), AJCC stage (P < 0.001), and pN stage (P < 0.005) as independent factors (**Table 3**; **Supplementary Figure 1B**).

**Table 3:** Univariate and multivariate analyses of cancer‐specific survival in the training cohort.

Factor	Univariate analyses		Multivariate analyses	
	*p* Value	HR (95% CI)	*p* Value	HR (95% CI)
**Sex**	0.474		0.519	
Male		Reference		Reference
Female		0.96(0.87-1.07)		0.97(0.87-1.07)
**Age**	**<0.001**		**<0.001**	
≤60 years		Reference		Reference
>60 years		1.57(1.41-1.73)		1.46(1.31-1.62)
Race				
Black		Reference		Reference
White	**0.473**	0.94(0.79-1.12)	**0.155**	0.88(0.74-1.05)
Chinese	**<0.001**	0.40(0.33-0.48)	**<0.001**	0.46(0.37-0.56)
Other	**<0.001**	0.61(0.48-0.76)	**0.004**	0.71(0.56-0.90)
Site				
1/3 U		Reference		Reference
1/3 M	**<0.001**	0.67(0.55-0.81)	0.741	0.96(0.77-1.20)
1/3 L	**<0.001**	0.59(0.52-0.68)	0.224	0.90(0.77-1.06)
Curvature	**<0.001**	0.52(0.44-0.61)	0.207	0.89(0.73-1.07)
Total stomach	**0.004**	1.28(1.08-1.51)	**0.003**	1.35(1.10-1.64)
others	**<0.001**	1.55(1.27-1.88)	**0.034**	1.27(1.02-1.60)
**Size**	**<0.001**		**<0.001**	
≤5 cm		Reference		Reference
>5 cm		3.01(2.71-3.35)		1.93(1.72-2.16)
Grade (%)				
I		Reference		
II	0.989	0.99(0.24-4.04)		
III	0.216	2.40(0.60-9.61)		
IV	0.189	2.62(0.62-11.02)		
NA	0.105	3.18(0.79-12.86)		
AJCC stage				
IA		Reference		Reference
IB	**0.004**	1.52(1.15-2.01)	**<0.001**	1.91(1.35-2.70)
IIA	**<0.001**	2.17(1.70-2.77)	**<0.001**	2.94(1.90-4.54)
IIB	**<0.001**	3.37(2.71-4.19)	**<0.001**	4.85(2.97-7.91)
IIIA	**<0.001**	4.23(3.42-5.24)	**<0.001**	6.06(3.35-10.95)
IIIB	**<0.001**	5.35(4.34-6.59)	**<0.001**	8.66(4.64-16.18)
IIIC	**<0.001**	6.89(5.64-8.42)	**<0.001**	12.55(5.94-26.53)
pT stage				
T1		Reference		Reference
T2	**0.001**	1.46(1.16-1.83)	0.078	0.74(0.53-1.03)
T3	**<0.001**	3.37(2.85-3.98)	0.317	0.82(0.56-1.21)
T4	**<0.001**	4.76(4.05-5.61)	0.211	0.74(0.46-1.19)
pN stage				
N0		Reference		Reference
N1	**<0.001**	1.94(1.68-2.24)	0.173	0.87(0.71-1.06)
N2	**<0.001**	1.91(1.63-2.24)	**<0.001**	0.62(0.47-0.81)
N3	**<0.001**	2.91(2.55-3.30)	**0.002**	0.58(0.42-0.82)
Therapy				
surgery only		Reference		
surgery plus neo/chemo	0.155	0.65(0.54-0.78)		
surgery plus chemo	0.133	0.92(0.81-1.03)		
surgery plus neo	0.981	1.00(0.81-1.24)		
NA	0.155	0.72(0.10-5.10)		

pN, pathologic N stage; pT, pathologic T stage; CI, confidence interval; HR, hazard ratio. NA, not applicable.

### The nomograms for OS and CSS were established

**Figure 2:**
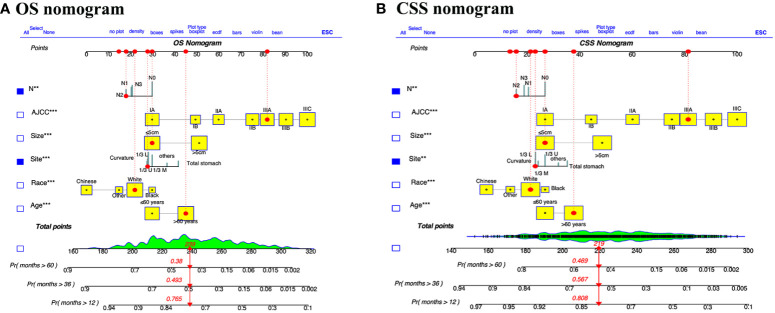
Dynamic nomograms for predicting the prognosis of patients with GSRCC. Nomograms for predicting the overall survival **(A)** and cancer specific survival **(B)** of GSRCC patients were created by integrating the six pivotal clinical prognostic factors. By drawing a vertical line straight upward from the factor’s associated parameter to the points axis, one may find the score for each risk factor. The survival probability of GSRCC patients after one, three, and five years after surgery can then be obtained by adding the scores of all risk factors together and drawing a straight line from the total points axis to the OS or CSS axis.

We integrated age, race, tumor size, tumor site, AJCC stage, and pN stage to develop comprehensive prognostic nomograms for evaluating the OS and CSS probabilities of patients with GSRCC, as depicted in **Figure 2**. The survival probabilities at 1, 3, and 5 years were computed graphically considering the individual patient’s unique characteristics, resulting in an interactive function. After converting the regression coefficients to a 0–100 point scale based on multivariate analysis. A vertical line is drawn from the value of the prognostic factor to the “Points” axis to determine the risk points associated with each prognostic factor. Subsequently, a vertical line is traced from the “Total Points,” representing the accumulation of risk points toward the final three axes, displaying the 1-year, 3-year, and 5-year survival rates, respectively, to determine the OS and CSS probability for a specific patient. To use the nomograms to predict the prognosis of an individual GSRCC patient, first determine the score for every variable based on the value on the topmost point row corresponding to its parameter.

**Figure 3:**
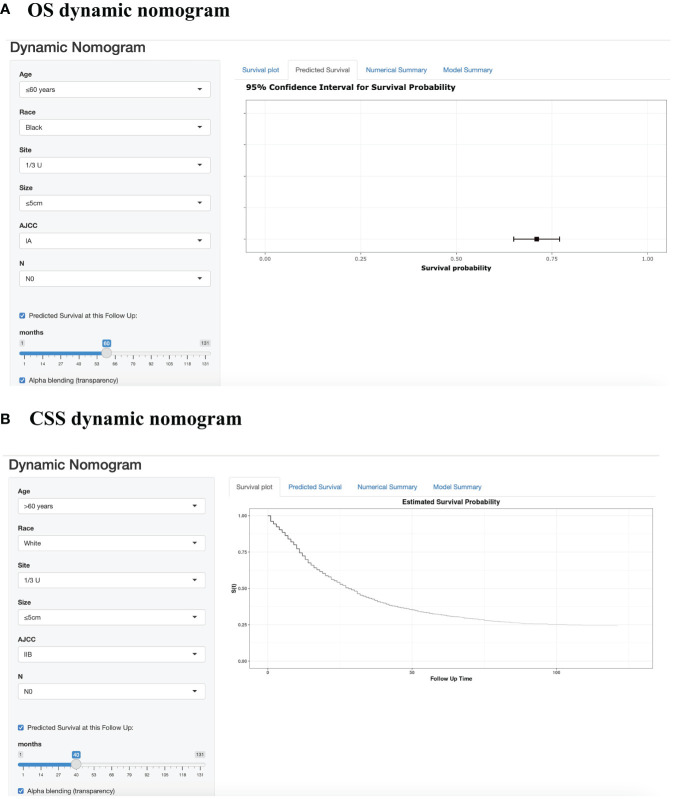
Web-based prognostic nomogram for patients with GSRCC. (Available at https://jiangyujuan.shinyapps.io/OS-nomogram/. and https://jiangyujuan.shinyapps.io/DynNomapp-DFS/.) **(A)** Overall survival. **(B)** Cancer-specific survival.

For instance, we examined a patient of white ethnicity with upper gastric cancer (28 points) at TNM stage IIIA (82 points) and pN2 (18 points) with a tumor size ≤ 5 cm (30 points) and aged > 60 years (45 points). Consequently, the total number of risk points is 173, and the survival probability axis can be determined by drawing a vertical line. The 1-, 3-, and 5-year survival probabilities of the patients were 90.0%, 72.7%, and 68.0%, respectively. The length of each variable line in these nomograms indicates its contribution to prognosis. For instance, our nomograms showed that AJCC stage had the most prominent impact on both CSS and OS in GSRCC patients among the included clinical parameters. Furthermore, we developed a dynamic web application that utilizes the constructed nomograms (**Figure 3**). Hyperlinks (https://jiangyujuan.shinyapps.io/OS-nomogram/ and https://jiangyujuan.shinyapps.io/DynNomapp-DFS/) can be accessed.

### Validation of the protein-associated prognostic model

**Figure 4:**
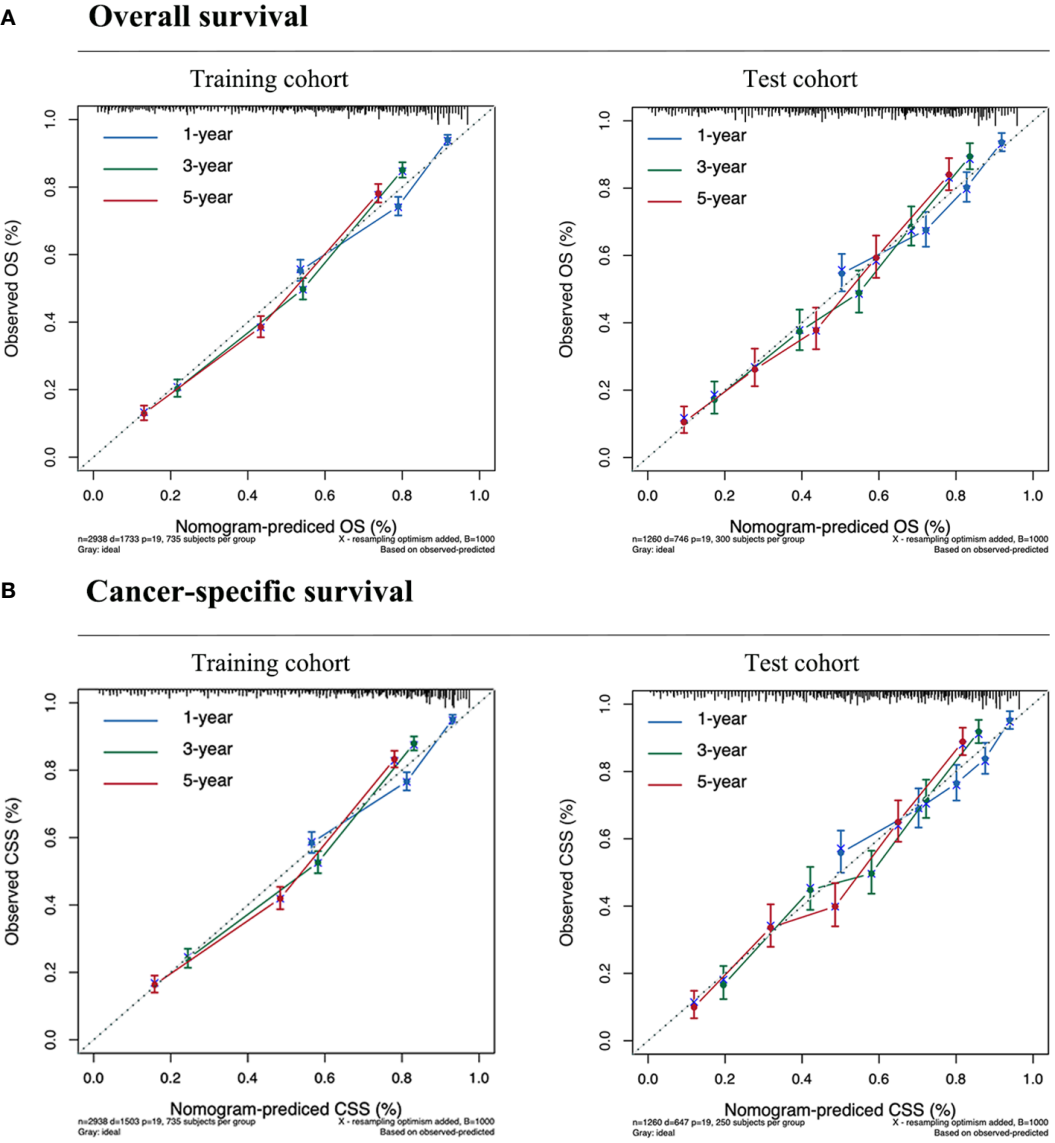
Calibration curves for predicting the survival of GSRCC patients. **(A)** Overall survival. **(B)** Cancer-specific survival. Our nomogram is represented by the solid line, while the ideal nomogram is represented by the 45-degree dotted line. The forecast is accurate if it falls on the 45-degree diagonal for the expected survival probability. The blue, green, and red lines represent 1, 3, and 5-year survival rates, respectively.

**Figure 5:**
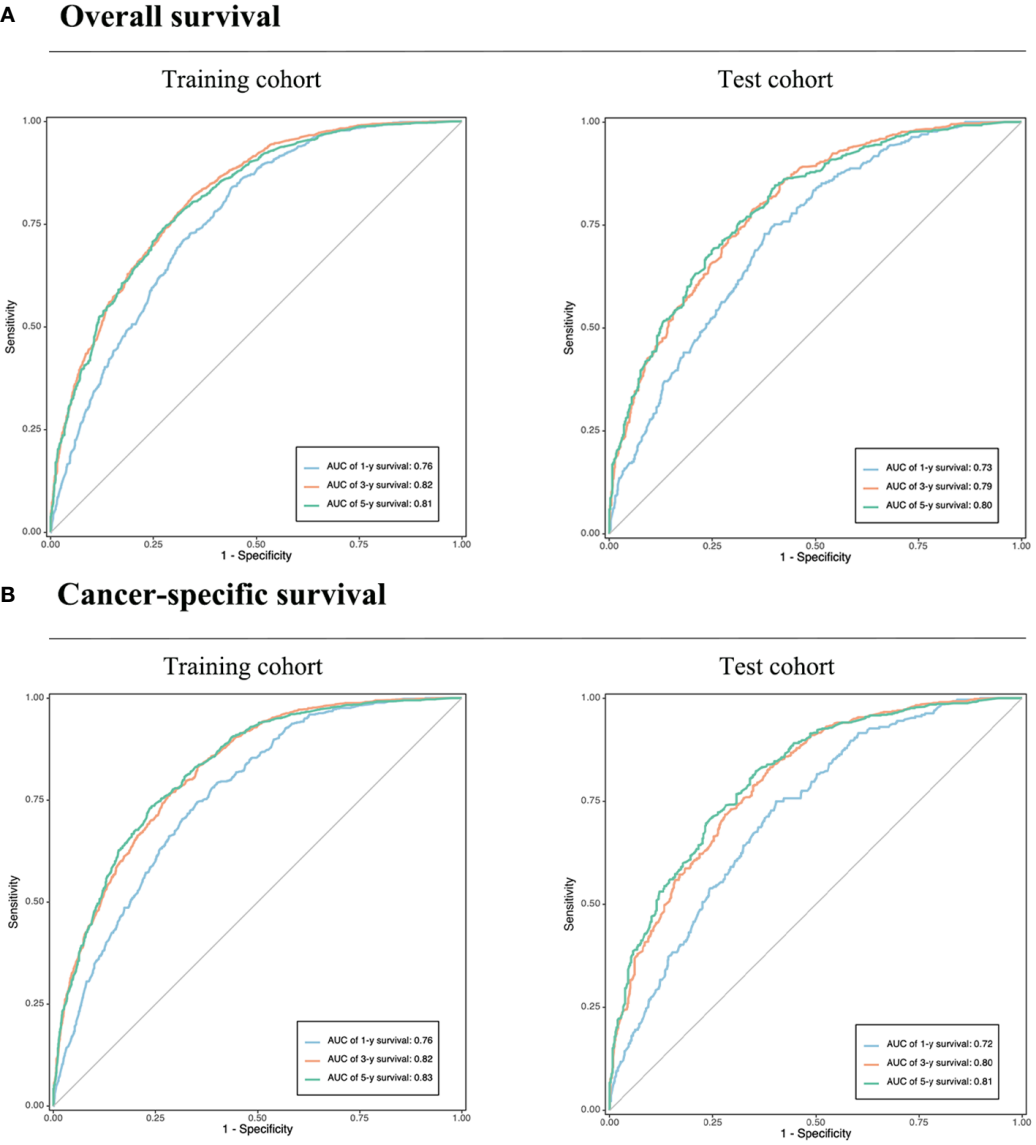
Validation of the prognostic nomograms using ROC curves. **(A)** Overall survival. **(B)** Cancer-specific survival. AUC, area under the curve. The blue, yellow, and green lines represent 1, 3, and 5-year survival rates, respectively.

The calibration plot, concordance index (c-index), area under the curve (AUC), and DCA curve were used to evaluate the performance of the predictive models in both the training and validation cohorts. Initially, calibration curves were constructed, revealing a close alignment between the actual outcomes of GSRCC patients in the training and validation cohorts and the 1-, 3-, and 5-year OS and CSS probabilities predicted by the nomogram models. These results indicated a high level of predictive accuracy (**Figure 4**). Second, the nomogram demonstrated favorable accuracy in predicting survival, as evidenced by c-index values of 0.735 ± 0.012 and 0.743 ± 0.012 for OS and CSS, respectively, in the training cohort, and 0.715 ± 0.019 and 0.719 ± 0.020, respectively, in the validation cohort. ROC curves were generated to assess the predictive sensitivity and specificity of the nomogram prediction models. For the training cohort, the 1-, 3-, and 5-year area under the curve (AUC) values for OS were 0.76, 0.82, and 0.81, respectively, and those for CSS were 0.76, 0.82, and 0.83, respectively. The area under the curve (AUC) values for the prediction models were consistently above 0.70 in the validation cohort (**Figure 5**).

**Figure 6:**
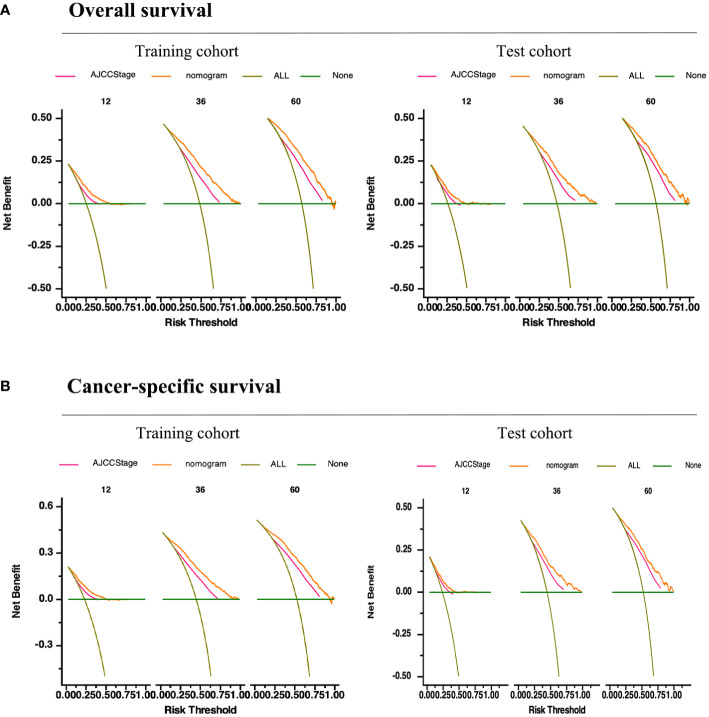
Validation of the prognostic nomograms using DCA curves. **(A)** Overall survival. **(B)** Cancer-specific survival. All, all the patients died or relapsed; None, no patients died or relapsed. The pink line represents the TNM staging and the yellow curve represents our prediction model.

In summary, these findings substantiate the relatively high sensitivity and specificity of our nomogram models. Furthermore, DCA has frequently been employed to assess the clinical utility of nomograms. The nomograms outperformed conventional TNM staging and demonstrated a substantial positive net benefit in terms of mortality risk, as depicted in **Figure 6**. These findings indicate the significant clinical utility of nomograms in predicting the OS and CSS of patients with GSRCC.

### Performance of the dynamic nomogram in stratifying patient risk status

**Figure 7:**
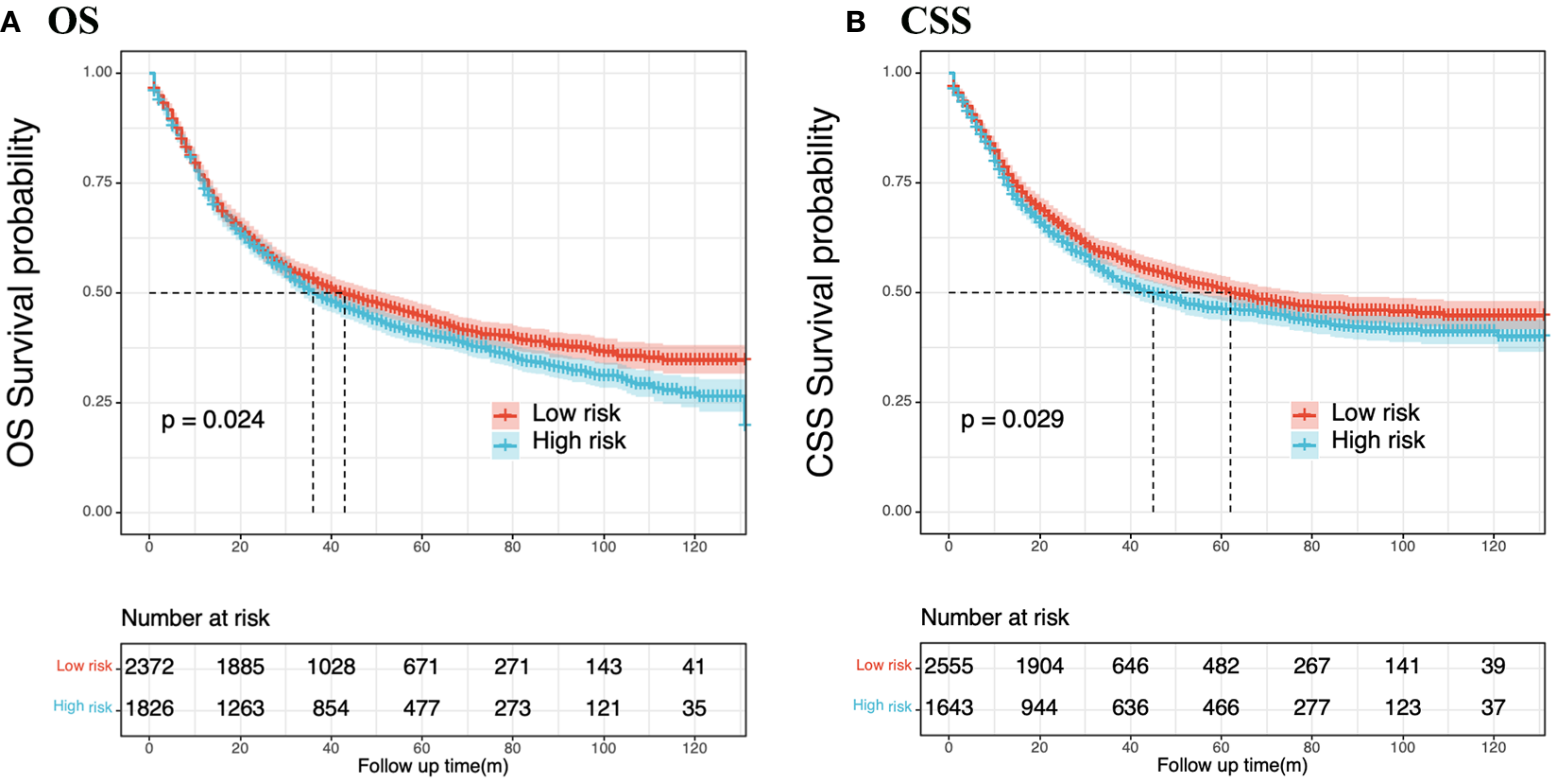
Nomogram-based risk stratification. GSRCC patients were divided into low- and high-risk subgroups by the nomogram score, and Kaplan-Meier survival analysis was performed to verify the clinical significance of the nomogram models. **(A)** Overall survival; **(B)** Cancer-specific cancer.

Predictor variable scores were calculated using the nomogram and then combined to determine the total scores for individual patients. Patients with GSRCC were categorized into low- and high-risk groups based on their nomogram scores, using a threshold value of 125.0 points for the CSS nomogram and 157.0 points for the OS nomogram. Patients with scores exceeding the threshold were assigned to the high risk group. Survival analysis revealed that the probabilities of CSS (*P* = 0.029) and OS (*P* = 0.024) were significantly lower in the high-risk group than in the low-risk group, indicating the potential of these nomograms for risk stratification in GSRCC patients (**Figure 7**).

## Discussion

GSRCC is a heterogeneous malignancy with a high risk of recurrence and death. For recurrent and metastatic GSRCC, there are currently few effective treatment options. To identify high-risk patients and implement early intervention and tailored treatment, it is crucial to create efficient prognostic prediction models. This study used data from the public SEER and NCC cohorts to develop and validate a predictive nomogram model for estimating the OS rate and CSS in patients with GSRCC. The nomograms incorporated six prognostic variables: age, race, tumor size, tumor site, N stage, and AJCC stage. Furthermore, a dynamic web application was developed to facilitate clinical decision making using these nomograms. The calibration of the nomograms demonstrated strong performance, with internal and external validations confirming their reliability. The OS and CSS nomograms also exhibited a C-index and AUC exceeding 0.7, indicating their effective discriminatory capability. Moreover, the decision curve analysis illustrated that our novel nomogram models provided a more significant net clinical benefit than the AJCC staging system across various threshold probabilities. These findings suggest that our nomograms could aid in developing tailored therapeutic strategies for the more effective treatment of patients with GSRCC.

This study focused on the GSRCC nomogram because of its controversial prognosis. Compared to other types of GC, GSRCC has unique tumorigenic properties and atypical epidemiological distribution (9). Zu et al. reported advanced GSRCC has a poorer prognosis than the other advanced gastric adenocarcinoma subtypes (10). The current staging system developed by the AJCC staging system for evaluating the prognosis of patients with AJCC staging system, cannot be used to effectively monitor GSRCC.

Only a few studies have revealed the prognostic factors of patients with GSRCC, and the related prognostic prediction models have been developed, although the models remain imperfect. For instance, the GSRCC prediction model that was presented by Liu et al. (5) and Zhang et al. (7) did not predict CSS; instead, it only addressed the OS. Nie et al. (8) only used data from the SEER database; hence, it is challenging to evaluate its accuracy and viability because there are insufficient external validations. Furthermore, a number of important clinicopathological parameters that have a substantial impact on patient survival—such as age, sex, and treatment type—are not taken into consideration by the GSRCC monitoring prediction models that are now in use. Therefore, further investigation is necessary to examine the factors influencing the long-term survival of patients with GSRCC and to develop valuable predictive models tailored to GSRCC. In this study, we conducted univariate and multivariate Cox regression analyses using extensive clinical data to identify independent risk factors for OS and CSS in patients with GSRCC.

GSRCC is associated with advanced disease, with a higher incidence of patients at AJCC stage IV, more advanced T and N stages, and higher tumor grade. These findings are consistent with those of a previous study that reported a greater frequency of advanced-stage GSRCC than early-stage GSRCC (11). In line with the AJCC staging system, our newly developed nomogram demonstrated a significant influence of lymph node presence on predicting survival outcomes. Furthermore, the independent prognostic factors identified within the context of GSRCC included age, race, tumor size, and tumor site. Leveraging these variables as independent prognostic factors in a nomogram has the potential to enhance the predictive efficacy of the model. Previous reports have indicated that young patients with GSRCC with low-stage tumors who underwent radical surgery exhibited a more favorable prognosis than other GSRCC patients in terms of survival (12). Previous studies have indicated that older age and advanced tumor stage are associated with poorer OS. Ren et al. stated that age was the primary factor influencing survival, with individuals older than 74 years experiencing poorer survival than those younger than 45 years old (13). Chu et al. discovered that OS significantly deteriorates in patients older than 60 years (14). Our results are consistent with those of previous studies. Therefore, early detection, diagnosis, and treatment of tumors are crucial to enhance patient survival rates.

According to multivariate analysis, the identified optimal cutoff for tumor size was deemed a significant independent prognostic factor. Consequently, tumor size was incorporated into the nomogram. In a prior study, Im et al. reported that larger tumor size was an independent prognostic factor associated with poorer prognosis (15). A larger tumor size stimulates angiogenesis, leading to increased tumor cell proliferation. However, the underlying mechanism requires further investigation. Environmental factors, lifestyle, diet, and genetics significantly influence the development of gastric cancer. Wang et al. and Sun et al. reported that individuals of white and black ethnicities have poorer survival rates than individuals of other ethnic groups (16, 17). These findings are consistent with our results. Ethnic differences play an essential role in the occurrence and development of gastric cancer. In conclusion, these findings are consistent with those of the present study.

Nomograms are graphical tools that transform clinicopathological feature scores to predict the likelihood of clinical occurrence. Integrating patient data from other ethnic groups and cancer registries with the SEER database raises the possibility that this methodology can be universally used. According to previous research, nomograms offer a substantial likelihood of predicting the survival of patients with malignant tumors (18, 19), even surpassing the traditional TNM staging system (20). Currently, mature prognostic models for GSRCC that can be widely implemented in clinical practice are lacking. Our nomograms have the potential to be utilized for clinical and predictive assessments of patients with GSRCC, aiding individualized treatment planning.

The strength of our research lies in the two dynamic prediction models we successfully created and validated, one of which was used to predict GSRCC patients with CSS, and the other to forecast their OS. Additionally, we created two web-based predictive model applications. These devices will be put into practice by clinical surgeons and will be made convenient. Additionally, we included the SEER database and the NCC cohort. The two current prediction models are based on these two large databases, covering both Western and Eastern populations, so we believe that the models are universal and can predict the prognosis of gastric signet ring cell carcinoma in different populations to a large extent generalizability. Finally, our findings demonstrated that our nomograms had good clinical benefits and high discriminant and accurate predictive power. In addition, another outstanding advantage of our study is the simultaneous analysis of postoperative OS and CSS in patients with GSRCC. Currently, the majority of research has overlooked the examination of CSS in favor of concentrating more on the prognostic factors of postoperative OS in patients with GSRCC. CSS refers to death caused by a specific disease, and at this time, the concern about whether the cause of death is caused by a specific disease begins. If it is not due to a specific disease, it is not included in the outcome measures. It is a good indicator of the clinical benefit of a specific disease. In this work, we constructed prediction models based on both OS and CSS, which can assist physicians in recognizing the clinical factors affecting the postoperative survival of GSRCC patients globally, and also pay attention to the clinical prognostic factors that are actually associated with cancer.

However, our study had certain limitations. First, several important details, such as the surgical margin and technique, precise postoperative chemotherapy regimen and course, and patient’s medical conditions, were missing from our study. Second, the retrospective nature of this study is another drawback that could lead to a recollection bias. Third, we excluded patients whose variables had uncertain data, to prevent selection bias. Further prospective studies are warranted in the future. Finally, another limitation of our study is that it only analyzes common clinicopathological factors and does not include molecular markers related to gastric cancer. In future studies, we will further incorporate molecular markers of gastric cancer such as Her-2, PD-1, and claudin18.2 to further enhance our prognostic prediction models.

In summary, using data from two sizable cohorts, we developed and validated two postoperative web-based nomograms to predict the 1-, 3-, and 5-year OS and CSS of patients with stage I–III GSRCC. We verified the great discriminating power, good consistency, and high clinical availability of the nomogram by comparing it with the AJCC staging system. The prediction models may offer useful prognostic data, such as a patient’s probability of death and recurrence, making it easier to treat GSRCC with precision and individualization. This approach will assist physicians in managing patients with GSRCC after surgery. Nevertheless, the performance of the models needs to be validated by multicenter prospective studies in the future.

## Data availability statement

The raw data supporting the conclusions of this article will be made available by the authors, without undue reservation.

## Ethics statement

The studies involving humans were approved by ethics committee of the National Cancer Center/Cancer Hospital, the Chinese Academy of Medical Science, and Peking Union Medical College. The studies were conducted in accordance with the local legislation and institutional requirements. The participants provided their written informed consent to participate in this study.

## Author contributions

YJ: Conceptualization, Writing – original draft. HH: Data curation, Writing – original draft. XS: Conceptualization, Writing – original draft. WL: Conceptualization, Methodology, Writing – original draft. YL: Conceptualization, Software, Writing – original draft. JL: Funding acquisition, Writing – review & editing. YT: Funding acquisition, Writing – review & editing.

## Supplementary material

Supplementary Figure 1 Forest plots of multivariate Cox regression analysis for OS and CSS in patients with GSRCC.

Supplementary Figure 2 Pearson’s correlation analysis was used to determine the correlations between the variables.
