# Supplementary figures and images for: A novel web-based dynamic prognostic nomogram for gastric signet ring cell carcinoma: a multicenter population-based study
Source: Front Immunol. 2024 Apr 10;15:1365834. doi: 10.3389/fimmu.2024.1365834 (PMC11039906; doi:10.3389/fimmu.2024.1365834)

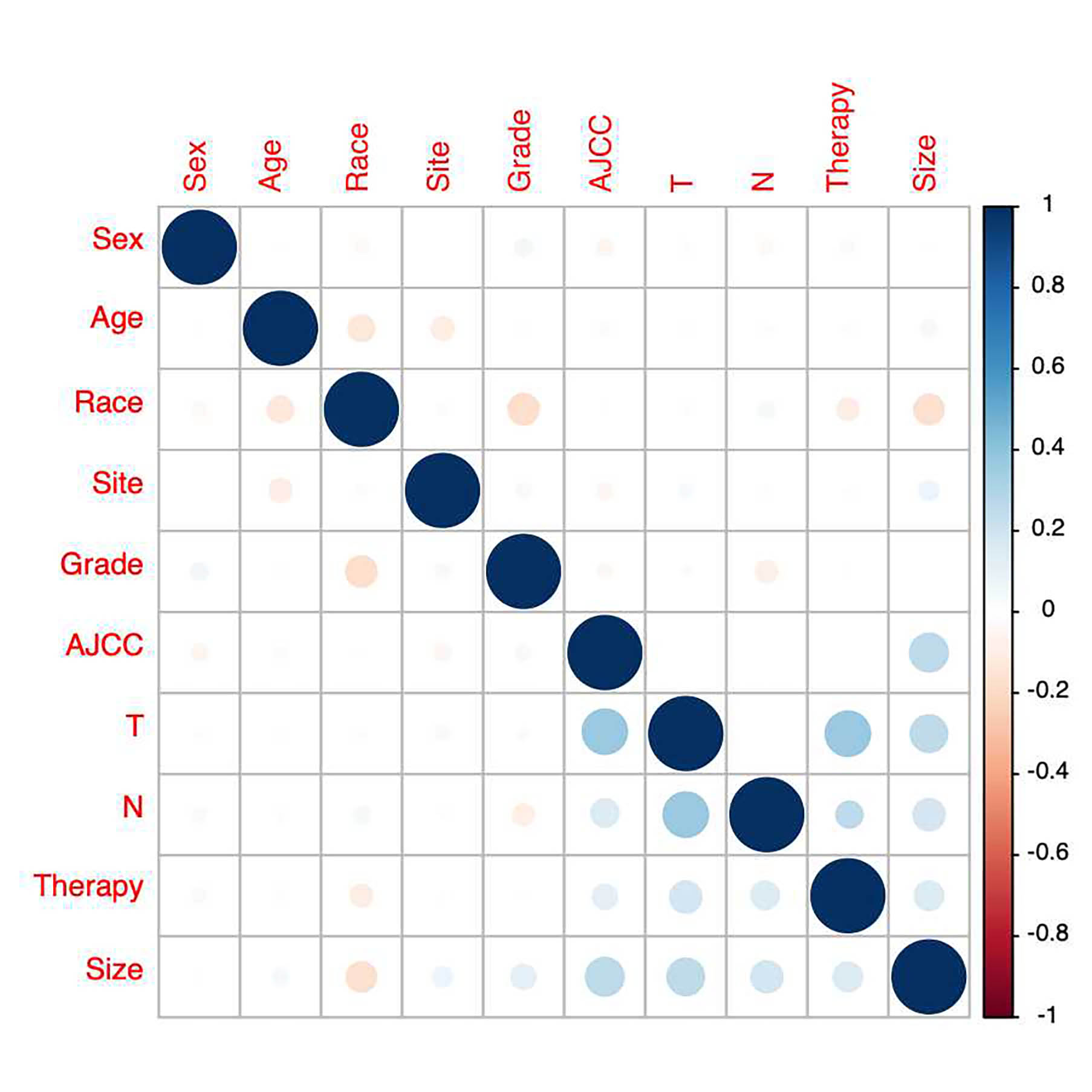

Supplement: Supplementary Figure 1 — Forest plots of multivariate Cox regression analysis for OS and CSS in patients with GSRCC. [file Image_1.jpeg]

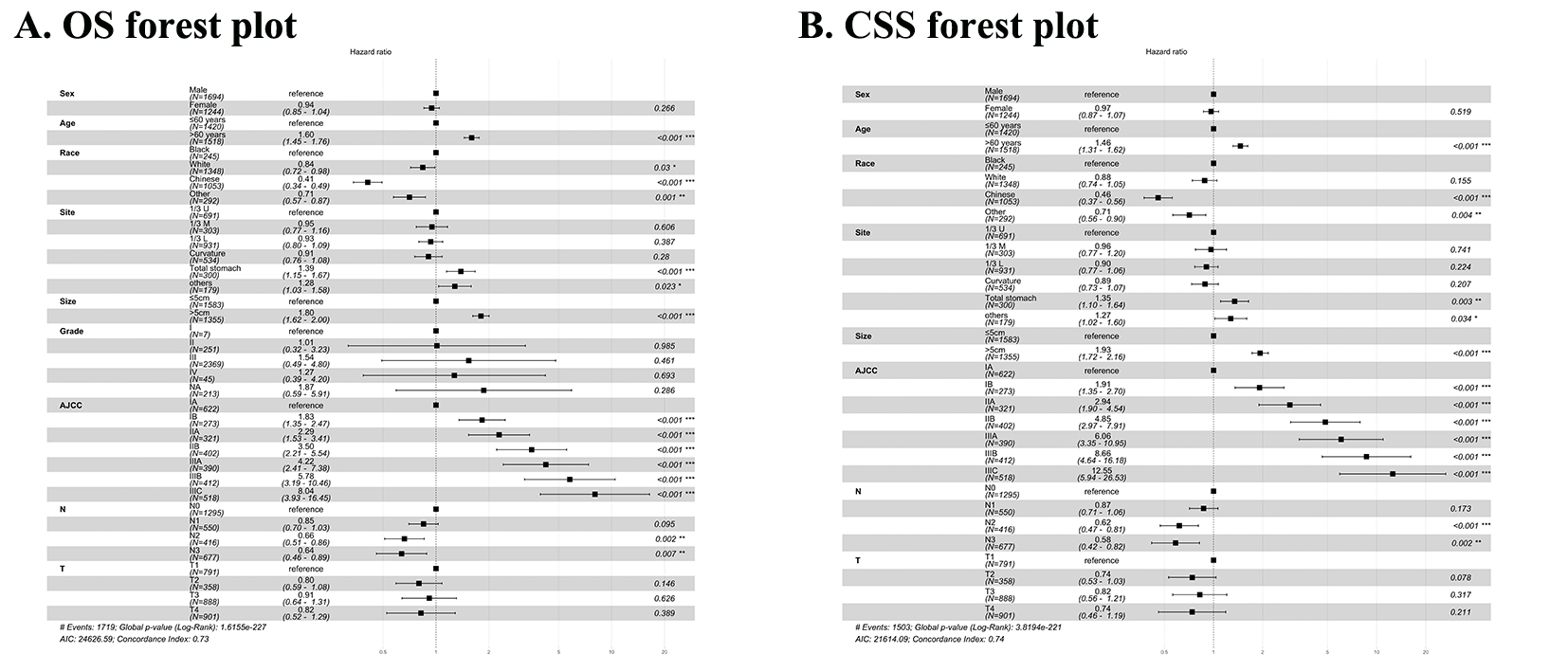

Supplement: Supplementary Figure 2 — Pearson’s correlation analysis was used to determine the correlations between the variables. [file Image_2.jpeg]
